# A Rare Case of Non-ossifying Fibroma Causing Pathological Subtrochanteric Femur Fracture in a Child: A Common Lesion at an Uncommon Location

**DOI:** 10.7759/cureus.73761

**Published:** 2024-11-15

**Authors:** Harsha G R, Satish Kumar, Harish M, Santhosh Kumar Tammali, Suhi Prakash Bharadwaj

**Affiliations:** 1 Orthopedic Surgery, Sri Siddhartha Medical College and Hospital, Tumakuru, IND; 2 Orthopedic Surgery, Acharya Shree Bhikshu Hospital, New Delhi, IND; 3 Orthopedic Surgery, Atal Bihari Vajpayee Institute of Medical Sciences and Dr. Ram Manohar Lohia Hospital, New Delhi, IND; 4 Orthopedic Surgery, Mahavir Institute of Medical Sciences and General Hospital, Vikarabad, IND

**Keywords:** lytic bone lesion, non-ossifying fibroma, paediatric orthopedics, pathological femoral shaft fracture, philos plating, subtrochanteric femur fracture

## Abstract

Non-ossifying fibroma (NOF) is a benign expansive lytic lesion more frequently found in children and adolescents at the metaphysis of long bones in and around the knee joint, typically resolving spontaneously or by ossification. This report presents a rare case of an 11-year-old child with a pathological subtrochanteric femur fracture attributed to an underlying NOF. We describe the diagnosis and surgical management of curettage and internal fixation using a proximal humerus locking plate, resulting in complete resorption of the lytic zone and an excellent clinical outcome. This report emphasizes recognizing benign lesions such as NOF at rare locations such as the proximal femur as potential contributors to pathological fractures in the pediatric population.

## Introduction

Non-ossifying fibromas (NOF) are one of the most common benign fibrous lesions characteristically found in children and adolescents. These lesions many a time are located around the knee, particularly in the distal femur and proximal tibia [1,2]. According to the WHO classification, they are considered a developmental anomaly [3] and typically resolve as the child enters adulthood [4]. Thus, finding NOF in the proximal femur further resulting in a pathological fracture is very rare. However, in some cases, these lesions can become large enough to compromise the integrity of the bone, leading to pathological fractures.

Subtrochanteric femur fractures are rarely found in the pediatric population and also pose a challenge in terms of the treatment plan and implant selection for open reduction and internal fixation (ORIF) [5,6]. To the best of our knowledge and a thorough search of the literature in the English language, there have been no reports of NOF causing subtrochanteric femur fracture. Here, we present a case of pathological subtrochanteric femur fracture in a child through an underlying NOF that was managed by curettage and ORIF with a proximal humerus locking plate achieving complete resorption of the lytic lesion and bone formation with good clinical outcomes.

## Case presentation

**Figure 1:**
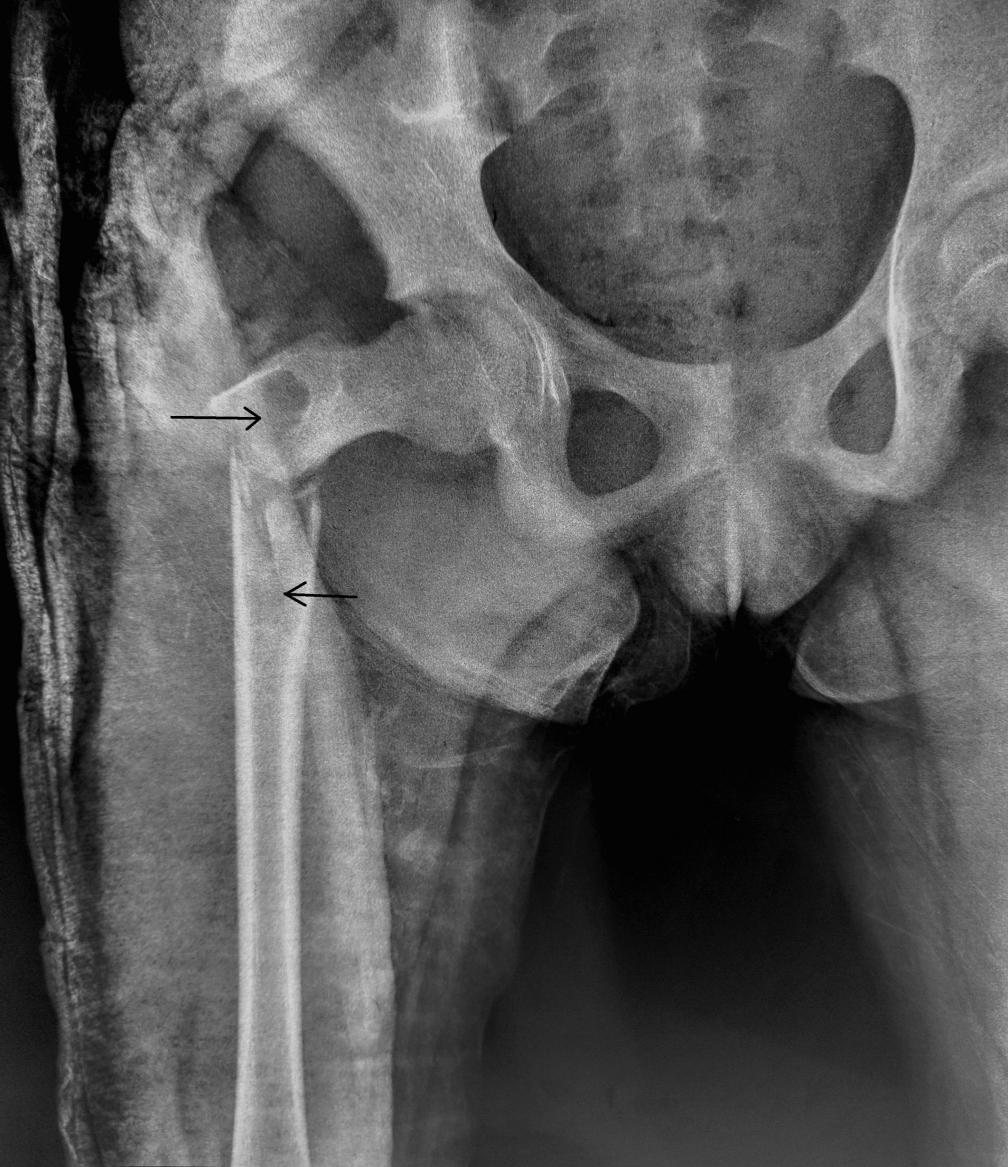
Preoperative X-ray of right hip (anteroposterior view) showing subtrochanteric femur fracture with a zone of lysis (black arrows) at the fracture site

A seemingly healthy 11-year-old girl was brought to the Emergency Room with extreme pain and an inability to bear weight on her right lower limb after she had a trivial fall while playing on her school grounds. Her limb was immediately splinted with a long leg plaster slab to help with the pain and deformity. X-ray of the right hip with femur was done and found to have a subtrochanteric femur fracture with intact physis in the femoral head and greater trochanter. Finding a subtrochanteric femur fracture after a trivial fall was extremely concerning and doubtful, and after scrutiny of the X-ray, we found a zone of lysis of around 3-4 cm at the fracture site as seen in Figure 1.

**Figure 2:**
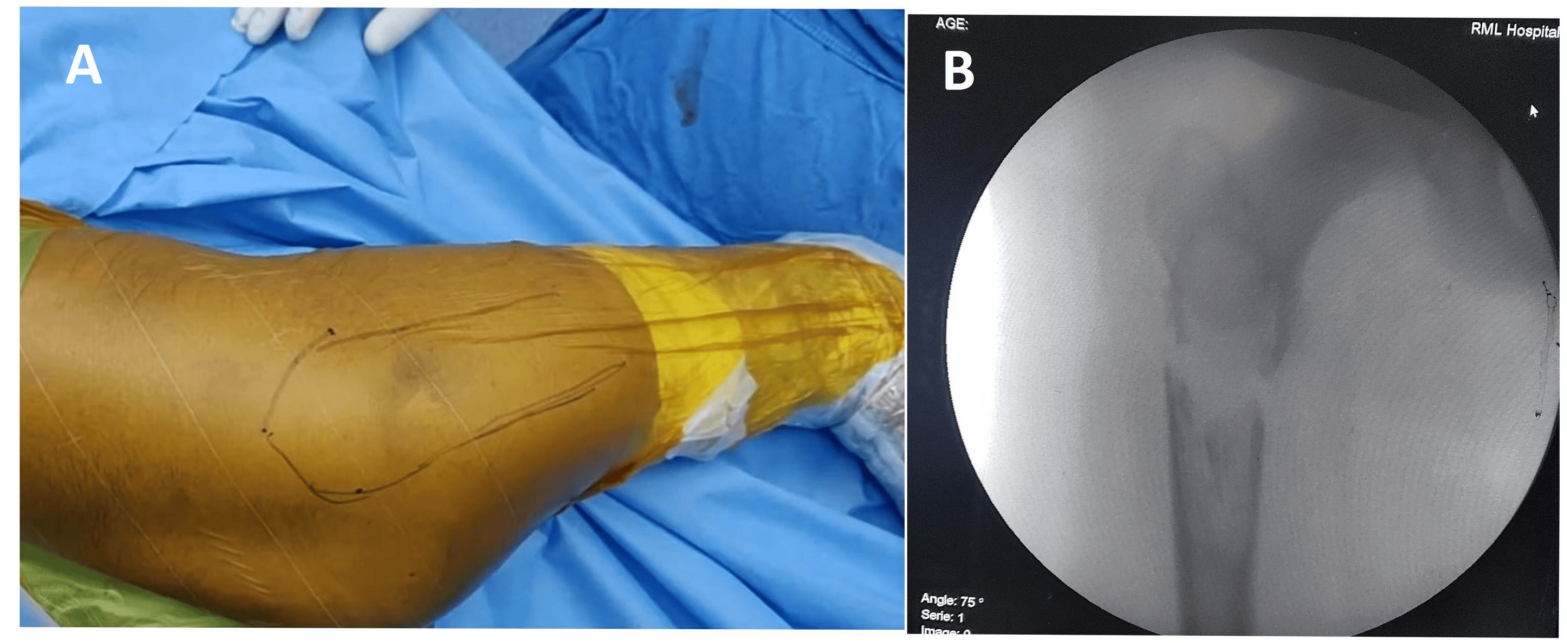
Intraoperative images showing (A) standard lateral approach to proximal femur being used; (B) On-table c-arm (anteroposterior view) showing lytic lesion of size around 2x3 cm while limb being held in traction

Although we insisted the patient go for a CT scan, due to financial restrictions, this was not done. Hence, we kept our differential diagnosis and proceeded with an open biopsy and curettage with ORIF with a polyaxial locking proximal humerus plate. The patient was placed in the left lateral position and approached with a standard lateral approach to the proximal femur. Once the fracture site was exposed, the lytic zone filled with hematoma was seen clinically and also confirmed under the c-arm (Figure 2).

The intraoperative biopsy sample was taken from the cavity and sent for histological examination. We then proceeded with thorough curettage followed by burning the wall of the cavity with cautery and hydrogen peroxide solution. Manual reduction of fracture site was done. Both anteroposterior and lateral alignment confirmed radiologically and fixed with a five-hole titanium proximal humerus polyaxial locking plate with three screws proximally in the femoral neck short of physeal line and four bicortical screws distally leaving the last screw hole empty.

Histological examination of the biopsy sample showed fibro-connective tissue entrapped in woven bone and surrounding inflammatory infiltrate suggestive of NOF.

**Figure 3:**
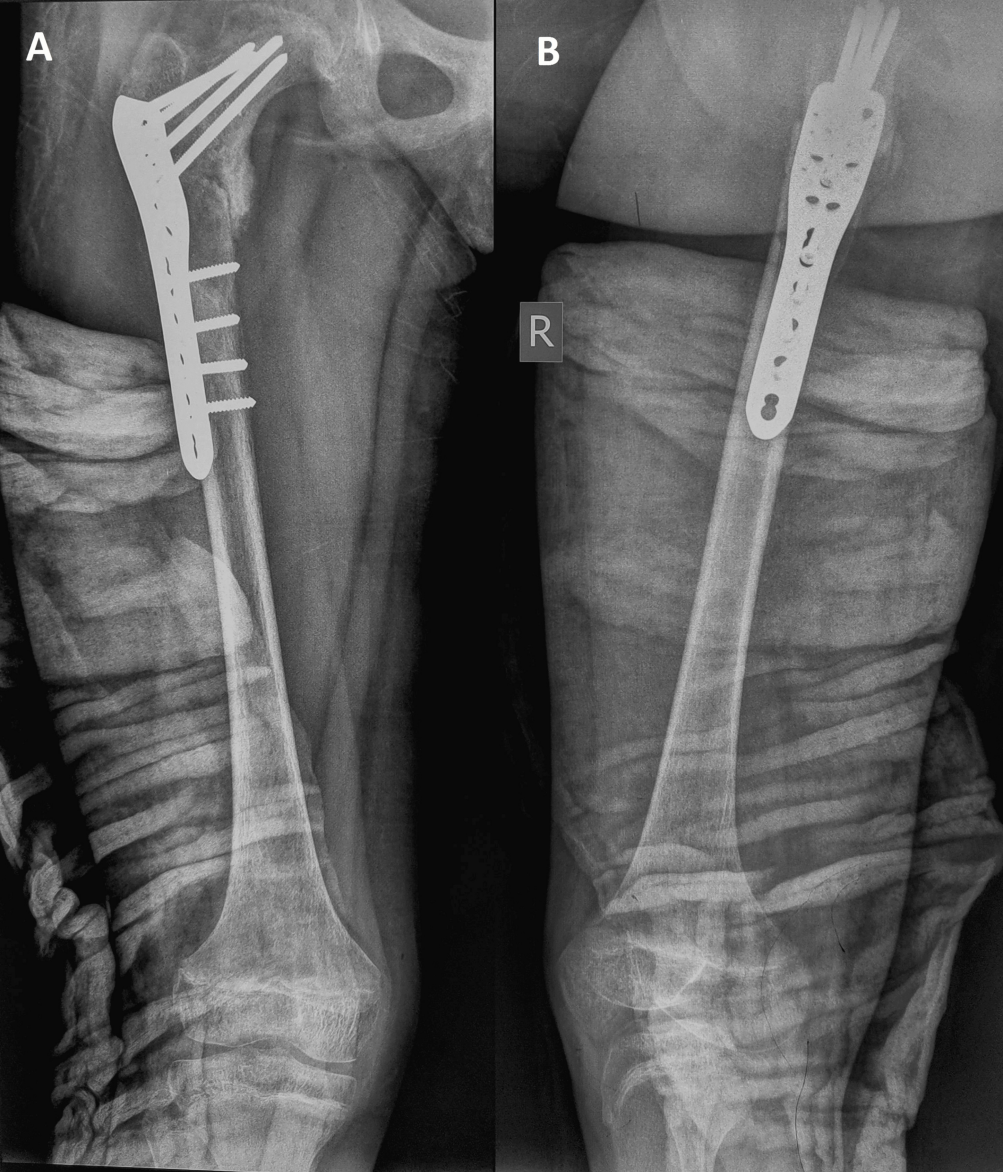
X-ray at six weeks postoperatively. (A) Anteroposterior view showing consolidation at fracture site; (B) Lateral view showing accurate placement of proximal screws within femoral neck

Post-fixation limb length was maintained adequately. The patient was placed in a long leg slab for a period of six weeks and encouraged to practice strict non-weight bearing with the help of a walker frame. The patient was put on oral antibiotic therapy for a week along with calcium and vitamin D supplementation. The first follow-up was done at six weeks where the X-ray showed gradual bone formation at the fracture site and consolidation at the lytic zone. The patient was then started with knee range of motion exercises and was asked to continue non-weight-bearing mobilization. During the eight-week postoperative period, the patient was started on partial weight-bearing mobilization on the affected limb (Figure 3).

**Figure 4:**
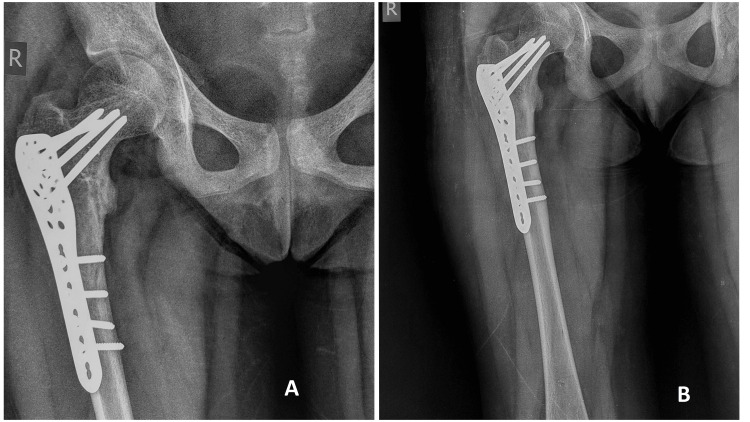
Postoperative images. (A) X-ray at eight weeks showing just a small line of fracture; (B) X-ray at 12 weeks depicts complete resorption of lytic lesion without any sign of previous fracture

The patient was started on full weight-bearing mobilization and hip range of motion exercises with a special emphasis on hip strengthening using isometric hip exercises followed by resistance band exercises once X-ray at 12 weeks showed a fully united subtrochanteric femur fracture with hardly any visible lytic zone. The last follow-up was done during a six-month post-operative follow-up in which the X-ray showed complete resorption of the lytic cavity without a sign of previous fracture (Figure 4).

**Figure 5:**
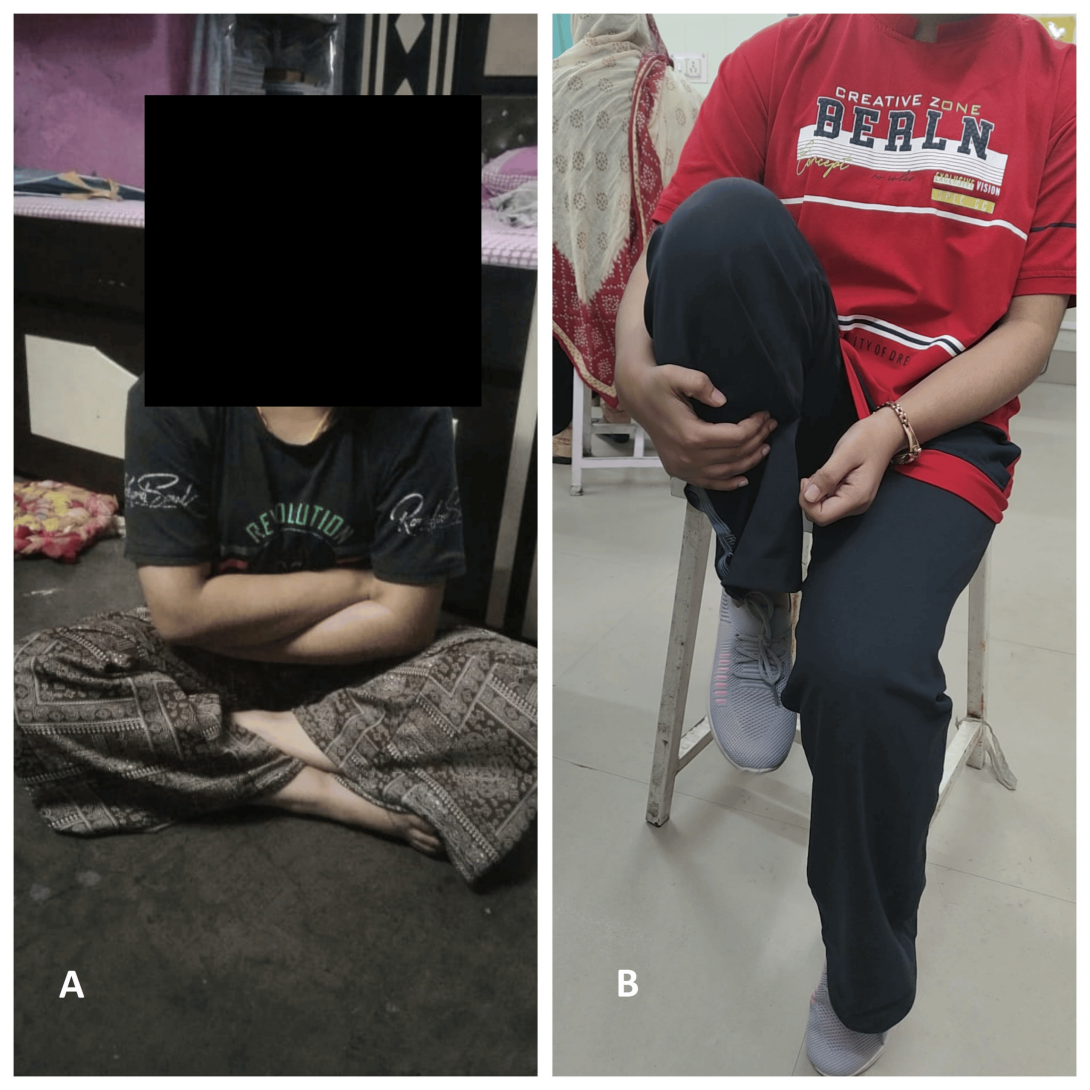
Functional outcomes. (A) Patient sitting comfortably in a cross-legged position from which she could stand without any support; (B) Patient showing complete hip flexion achieved which helped her to go back to cycling with ease

The patient was able to sit cross-legged without any pain by the end of six months and achieved complete functional activity without any complications. She was able to go back to her routine activities including cycling and running. The patient was advised to remove the plate later at the one-year postoperative follow-up (Figure 5).

## Discussion

NOFs are one of the most common benign focal lesions found in the pediatric population but are completely asymptomatic in up to 30% of the population in the first and second decades [1,5,6]. According to the WHO classification of tumors, NOF represents true benign neoplasms driven by activated mitogen-activated protein kinase (MAPK) signaling. Most lesions resolve with growing age; hence, many surgeons refer to NOF as ‘don't touch lesions’ since aggressive management is not required in such cases [7-9].

Radiologically, NOF may present as a metaphyseal eccentric focal lesion with thinned-out cortices rarely with any periosteal reaction found most often incidentally [10,11]. Diagnosis of NOF can be made with radiographs. Rammanohar et al., in their case, series emphasize the unnecessary usage of CT and MRI for the diagnosis of NOF by clinicians where only radiographs and proper history are sufficient for the diagnosis [11]. 

**Figure 6:**
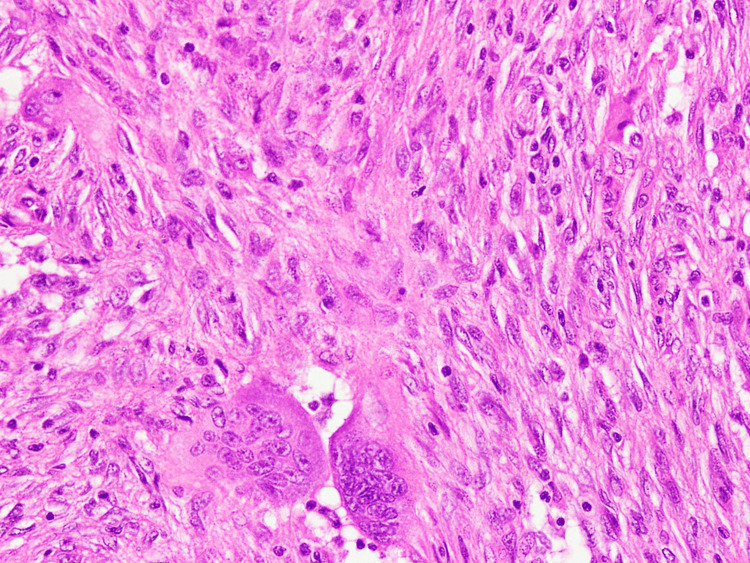
An example of histological slide (cross-section view) of non-ossifying fibroma (not of the current patient) Courtesy of: Nasir Ud Din, M.B.B.S., and PathologyOutlines.com [12]

An example of a histological slide showing NOF (not of the current patient) is shown in Figure 6. This is an H&E stained low power section showing the characteristic storiform arrangement of bland-looking spindle-shaped fibroblasts, with interspersed multinucleated osteoclast-like giant cells and some inflammatory cells [12].

According to the natural progression of these lesions, Ritschl et al. have proposed stages of lesion among which stage B with thinned-out cortices had the highest risk of fracture through the lesion [13]. Herget et al. also propose that stage B NOF is more amenable to fractures [14]. Thus, Arata et al. propose that follow-up and prophylactic fixation might be needed in cases where lesions exceed more than 50% of cortical diameter in both AP and lateral radiographs [15]. In the majority of cases, NOFs are typically found in and around the knee joint, the most common sites being the distal femur, proximal tibia, distal tibia, proximal humerus, fibula, and radius, and rarely even in the mandible [14-18]. However, throughout the literature, there isn’t a single description of any case of NOF in the proximal femur causing pathological subtrochanteric femur fracture like the one we are reporting.

Pediatric subtrochanteric femur fractures are often associated with high-velocity injuries [5,19]. If it is found after a trivial fall, there is a very high chance that it is pathological. Pathological subtrochanteric femur fractures cannot be managed conservatively like the ones in and around the knee joint since they require longer immobilization and difficult rehabilitation post-union [6,19,20]. Many studies suggest various implant options for internal fixation of subtrochanteric femur fracture, prominent among them are transcutaneous electrical nerve stimulation (TENS) and locking plates [5,6,18]. Since the fracture needed to be fixed along with curettage of the lesion and also keeping in mind not to breach the physeal lines, an adult proximal humerus polyaxial locking plate was chosen as described in multiple case reports with good clinical outcomes [19,21]. In all the reported cases of a pathological fracture through NOF, postoperative healing and rehabilitation were good with late complications like refracture, physeal arrest, and chronic pain [8,14]. This is the first accurate description of a NOF-associated pathological subtrochanteric femur fracture in a child which was managed effectively with curettage and proximal humerus plate fixation.

## Conclusions

This was a case of pathological subtrochanteric femur fracture caused by an underlying NOF, managed with curettage and ORIF with a proximal humerus locking plate with excellent radiological and functional outcomes achieving complete hip range of motion. Although extremely rare, clinicians should bear in mind that a NOF can occur at the proximal femur and also can present as a pathological subtrochanteric femur fracture. Thus, keeping NOF as a differential diagnosis in pathological subtroch femur fracture is warranted by this case report.
